# miR-29 Represses the Activities of DNA Methyltransferases and DNA Demethylases

**DOI:** 10.3390/ijms140714647

**Published:** 2013-07-12

**Authors:** Sumiyo Morita, Takuro Horii, Mika Kimura, Takahiro Ochiya, Shoji Tajima, Izuho Hatada

**Affiliations:** 1 Laboratory of Genome Science, Biosignal Genome Resource Center, Institute for Molecular and Cellular Regulation, Gunma University, Gunma 371-8512, Japan; E-Mails: msumiyo@gunma-u.ac.jp (S.M.); horii@gunma-u.ac.jp (T.H.); mikimura@gunma-u.ac.jp (M.K.); 2 Division of Molecular and Cellular Medicine, National Cancer Center Research Institute, 5-1-1, Tsukiji, Chuo-ku, Tokyo 104-0045, Japan; E-Mail: tochiya@ncc.go.jp; 3 Laboratory of Epigenetics, Institute for Protein Research, Osaka University, 3-2 Yamadaoka, Suita, Osaka 565-0871, Japan; E-Mail: tajima@protein.osaka-u.ac.jp

**Keywords:** cancer, DNA demethylation, microRNA, miR-29, TDG, TET1

## Abstract

Members of the microRNA-29 (miR-29) family directly target the DNA methyltransferases, DNMT3A and DNMT3B. Disturbances in the expression levels of miR-29 have been linked to tumorigenesis and tumor aggressiveness. Members of the miR-29 family are currently thought to repress DNA methylation and suppress tumorigenesis by protecting against *de novo* methylation. Here, we report that members of the miR-29 family repress the activities of DNA methyltransferases and DNA demethylases, which have opposing roles in control of DNA methylation status. Members of the miR-29 family directly inhibited DNA methyltransferases and two major factors involved in DNA demethylation, namely tet methylcytosine dioxygenase 1 (TET1) and thymine DNA glycosylase (TDG). Overexpression of miR-29 upregulated the global DNA methylation level in some cancer cells and downregulated DNA methylation in other cancer cells, suggesting that miR-29 suppresses tumorigenesis by protecting against changes in the existing DNA methylation status rather than by preventing *de novo* methylation of DNA.

## 1. Introduction

DNA methylation is a conserved epigenetic silencing mechanism that is involved in many important biological processes, including defense against transposon proliferation, control of genomic imprinting and regulation of transcription [1–3]. The DNA methylation status is modified in cancer [4]; aberrant patterns of DNA methylation are associated with tumor type, stage, prognosis and response to chemotherapy. Therefore, maintenance of the DNA methylation status is important for prevention of tumorigenesis. DNA methyltransferases (DNMTs) and DNA demethylases are responsible for the control of DNA methylation. DNMT3A and DNMT3B possess *de novo* methylation activity in mammalian cells [5,6], and mutations in DNMT3A occur in acute myeloid leukemia [7]. Active demethylation of DNA is mediated by thymine DNA glycosylase (TDG) and the ten-eleven translocation (TET) family of proteins [8]. In fact, TET1, TET2 and TET3 were recently identified as a new family of enzymes that alter the methylation status of DNA [9]. TET proteins are 2-oxoglutarate- and Fe(II)-dependent dioxygenases that catalyze the hydroxylation of 5-methylcytosine to 5-hydroxymethylcytosine and the subsequent generation of 5-formylcytosine and 5-carboxylcytosine, both of which are removed by TDG and base excision repair [10,11]. TET1 and TET2 are involved in tumorigenesis; TET1 is a fusion partner of the mixed lineage leukemia protein in acute myeloid leukemia and acute lymphoblastic leukemia, and loss-of-function mutations in the *TET2* gene are frequently observed in acute myeloid leukemia (AML), as well as a variety of myelodysplastic syndromes and myeloproliferative disorders [12]. A recent analysis of the exome in colon cancer also revealed mutations in the *TET1*, *TET2* and *TET3* genes [13].

The microRNA-29 (miR-29) family is implicated in epigenetic regulation, because DNMT3A and DNMT 3B are direct targets of miR-29 [14]. This miRNA family is also implicated in cancer; miR-29b suppresses prostate cancer metastasis by regulating epithelial-mesenchymal transition signaling pathways [15]. In addition, increased expression of miR-29a is associated with a longer disease-free survival period in stage II colon cancer patients [16], and lower levels of miR-29 expression are associated with shorter survival periods in mantle cell lymphoma patients [17]. Expression of the miR-29 family is also commonly downregulated in lung cancer [18,19].

The study of DNA demethylation is entering a period of rapid discovery, because DNA demethylases, such as TDG and members of the TET family, have recently been identified. Some DNA demethylases are now known to play important roles in biological phenomena and diseases that were previously poorly understood. Therefore, an understanding of the regulation of DNA demethylases is required. In this study, we report that the miR-29 family targets the 3′-UTRs of *TET1* and *TDG*.

## 2. Results and Discussion

### 2.1. Identification of miRNAs Targeting TET1 and TDG

**Figure 1:**
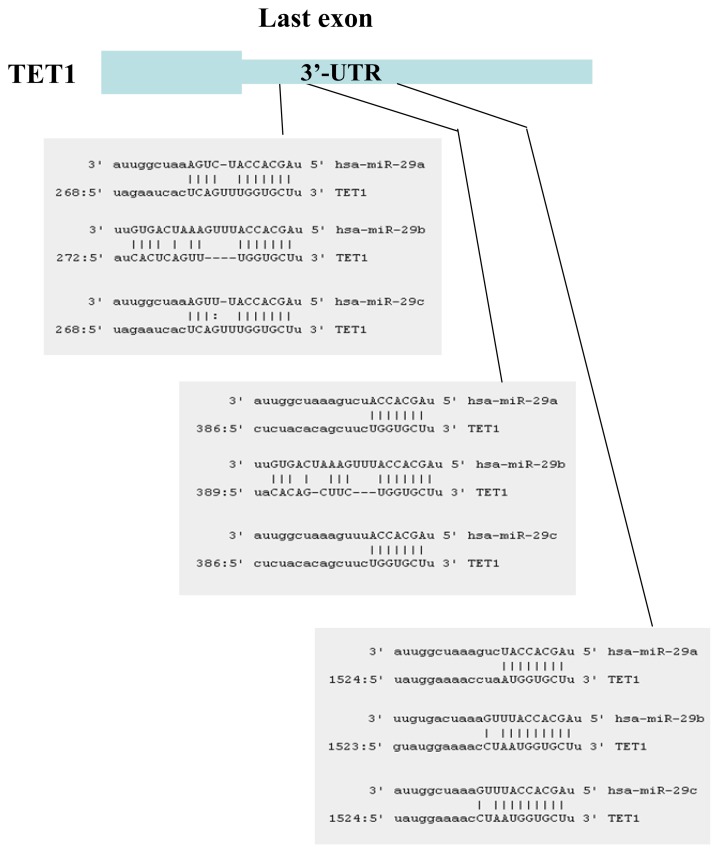
Three miR-29 target sites are present in the 3′-UTR of TET1.

**Figure 2:**
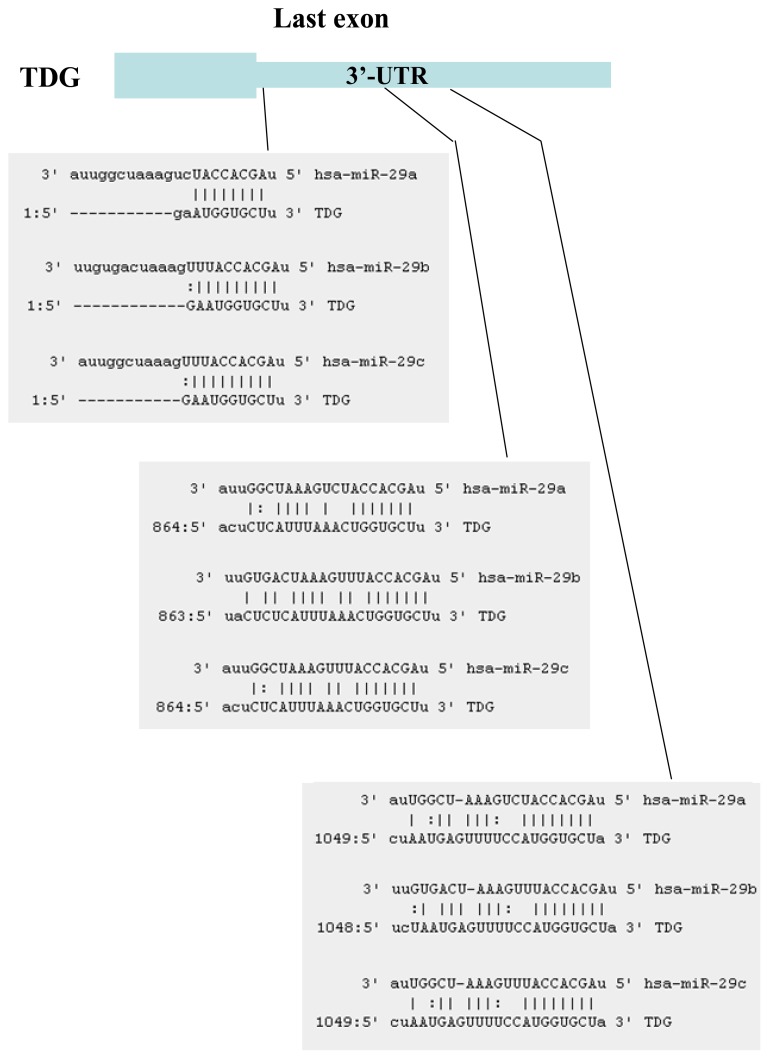
Three miR-29 target sites are present in the 3′-UTR of thymine DNA glycosylase (TDG).

The miRNAs that target TET1 and TDG, which are two key components of DNA demethylation, were identified using the target prediction software miRanda [20]. Although several miRNAs that target TET1 were identified, only six of these miRNAs were conserved between human and mouse: miR-29a, miR-29b, miR-29c, miR-590-3p, miR-376b and miR-653. Fourteen miRNAs that target TDG were identified: miR-29a, miR-29b, miR-29c, miR-186, miR-124, miR-30a, miR-30b, miR-30c, miR-30d, miR-30e, miR-26a, miR-26b, miR-410 and miR-495. We focused on the miR-29 family, because members of this family target both TET1 and TDG and because expression of miR-29 is downregulated in lung cancers [18,19]. TET1 (Figure 1) and TDG (Figure 2) were found to contain multiple target interaction sites for miR-29.

### 2.2. The miR-29 Family Targets and Regulates Expression of TET1 and TDG

**Figure 3:**
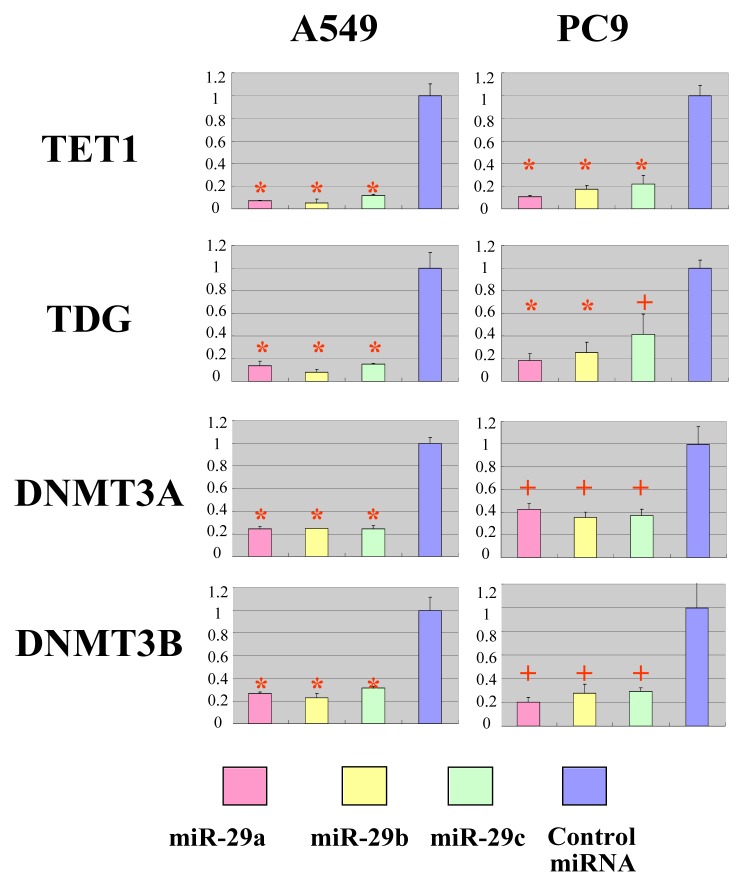
Members of the miR-29 family target the 3′-UTRs of TET1 and TDG. Luciferase activity of the TET1, TDG, DNMT3A and DNMT3B constructs was measured 48 h after cotransfection of A549 or PC9 cells with miR-29 or control miRNA. For each construct and cell line, data are normalized to the activity of cells transfected with control miRNA. Data show the mean + SD for *n* = 3 repeats. *p < 0.01, ^+^*p* < 0.05, compared with control miRNA.

**Figure 4:**
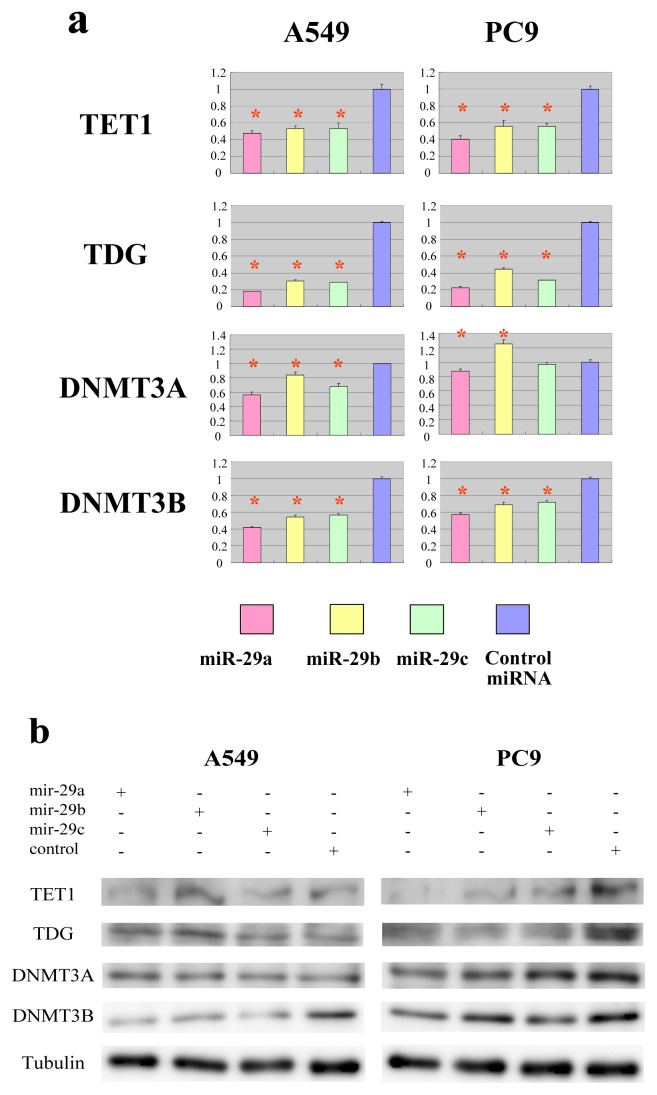
Members of the miR-29 family impact endogenous *TET1* and *TDG*. (**a**) Expression of mRNA. Quantitative RT-PCR analyses of the expression levels of *TET1*, *TDG*, *DNMT3A* and *DNMT3B* mRNAs were performed 48 h after transfection of A549 or PC9 cells with miR-29 or control miRNA. The actin gene was used to normalize the quantification of expression. Data are normalized to the mRNA expression in cells transfected with control miRNA. Data show the mean + SD for *n* = 3 repeats. *p < 0.01, compared with control miRNA; (**b**) Protein level. Western blots of TET1, TDG, DNMT3A and DNMT3B were performed 48 h after transfection of A549 or PC9 cells with miR-29 or control miRNA. The tubulin protein was used as endogenous control.

To validate the predicted interactions of TET1 and TDG with miRNA-29, chimeric constructs in which the TET1 or TDG 3′-UTR was inserted into the 3′-UTR of the firefly luciferase gene were generated, and the chimeric constructs were cotransfected with miR-29a, miR-29b, miR-29c or control miRNA into A549 and PC9 lung adenocarcinoma cell lines. Since DNMT3A and DNMT3B are known targets of miR-29, chimeric luciferase reporter constructs containing the miRNA-29 target sites in these genes were also generated, and parallel experiments were performed. Compared with the effect of the control miRNA, the three miR-29 species significantly reduced the luciferase activities of the TET1 and TDG constructs (Figure 3). As expected, the three miR-29 miRNAs also reduced the luciferase activities of the DNMT3A and DNMT3B reporter constructs (Figure 3) [14]. To investigate whether ectopic expression of miR-29 downregulates endogenous expression of *TET1* and *TDG* mRNAs, quantitative RT-PCR analyses of RNA extracted from A549 and PC9 cells transfected with miR-29a, miR-29b, miR-29c or control miRNA were performed. Compared with the effect of the control miRNA, transfection of cells with the three miR-29 constructs resulted in reduced expression of *TET1* and *TDG* mRNAs (Figure 4a). Expression levels of *DNMT3A* and *DNMT3B* mRNAs were also downregulated in A549 cells transfected with miR-29a, miR-29b or miR-29c (Figure 4a), as previously reported for this cell line [14]. Conversely, expression of *DNMT3A* mRNA was not downregulated in PC9 cells transfected with miR-29b (Figure 4a), whereas *DNMT3B* mRNA expression was downregulated in PC9 cells transfected with the miR-29 constructs (Figure 4a). We performed Western blot for TET1, TDG, DNMT3A and DNMT3B (Figures 4b and S1–S6). MiR-29s effectively reduced the protein levels of TET1 and TDG in PC9 cells, while not in A549 cells. On the other hand, MiR-29s could not effectively reduce the protein level of DNMT3A in both cells and only effectively reduced the protein level of DNMT3B in A549 cells, while not in PC9 cells. The discrepancy between the results of quantitative PCR and Western blot could be explained by the difference in stability of these proteins in each cell line. If TET1 protein is stable in A549 and unstable in PC9, the reduction of TET1 mRNA by miR-29 efficiently reduces TET1 protein level in PC9; however, TET1 protein level is not efficiently reduced in A549.

### 2.3. The miR-29 Family Regulates DNA Methylation

**Figure 5:**
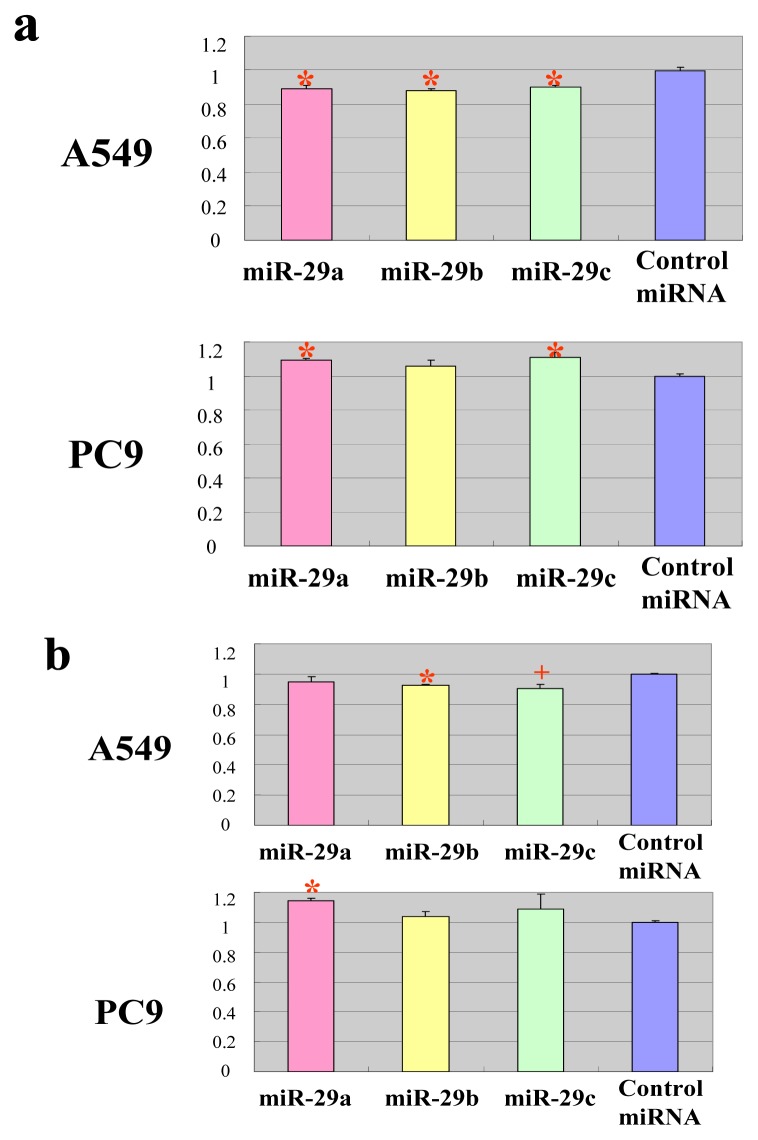
The DNA methylation level of cells transfected with miR-29 or control miRNA. (**a**) The global DNA methylation level. A luminometric methylation assay (LUMA) assay was performed 48 h after transfection of A549 or PC9 cells with miR-29a, miR-29b, miR-29c or control miRNA. Data are normalized to the DNA methylation ratio of cells transfected with control miRNA. Data show the mean + SD for *n* = 3 repeats. *p < 0.01, compared with control miRNA; (**b**) The DNA methylation level of RASSF1. A quantitative methylation-specific PCR of RASSF1 was performed. *p < 0.01, ^+^*p* < 0.05, compared with control miRNA.

Targeting of TET1 and TDG by miR-29 suggests that members of this miRNA family contribute to the regulation of DNA methylation in cancer. To address this hypothesis, A549 and PC9 cells were transfected with miR-29a, miR-29b, miR-29c or control miRNA, and then global DNA methylation levels were measured 48 h after transfection using a luminometric methylation assay (LUMA) [21]. Compared with the control miRNA, all three miR-29 constructs significantly reduced the level of global DNA methylation in A549 cells (Figure 5a), as previously reported [14]. This response is most likely attributable to repression of DNMT3B by miR-29. DNMT3A, whose protein level was not reduced in A549, might not be the major *de novo* DNA methyltransferase and might not significantly contribute to DNA methylation. On the other hand, miR-29a and miR-29c significantly upregulated global DNA methylation in PC9 cells (Figure 5a), which may be due to repression of TET1 and TDG by these miRNAs. Similar trends were also observed in the methylation of tumor suppressor RASSF1 gene (Figure 5b). One of the possible explanations for why A549 cells show decreased methylation by ectopic miR-29s, while PC9 cells acquire increased methylation levels, is that miR-29s were less effective on DNMT3 in PC9 cells than in A549 cells, while miR-29s were less effective on TET1 and TDG in A549 cells than in PC9 cells (Figure 4b), so that only demethylation was suppressed in PC9 cells and only methylation was suppressed in A549 cells. However, methylation differences are extremely low despite being significant; we should be extremely careful when drawing any conclusion from this experiment.

**Figure 6:**
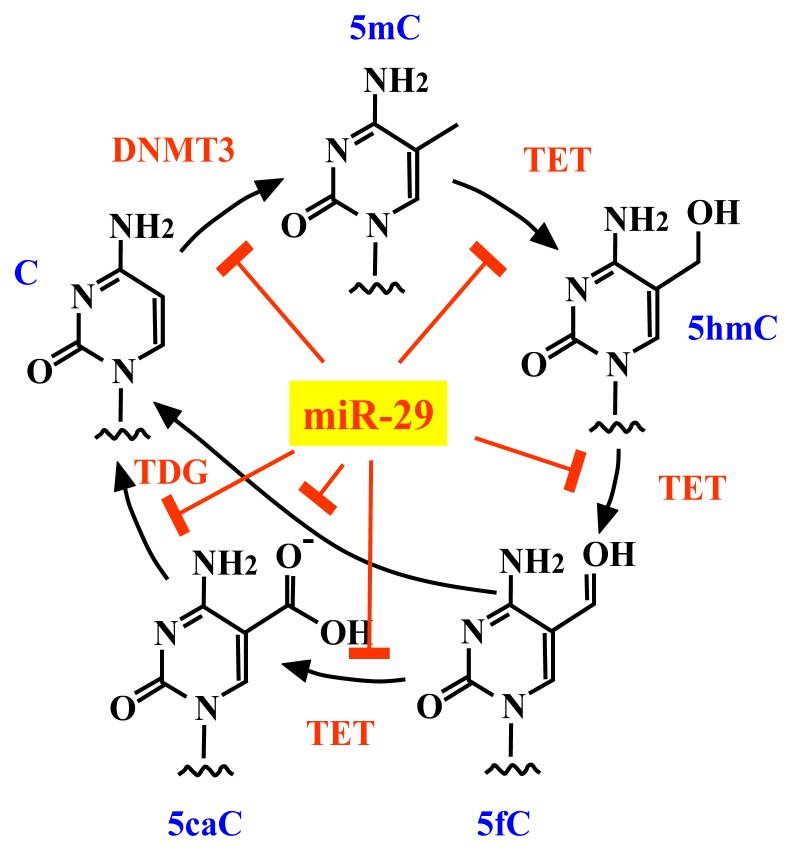
Regulation of DNA methylation by miR-29. Activities of DNMTs and DNA demethylases (TET, TDG) are repressed by miR-29. C, cytosine; 5mC, 5-methylcytosine; 5hmC, 5-hydroxymethylcytosine; 5fC, 5-formylcytosine; 5caC, 5-carboxylcytosine.

Members of the miR-29 family directly target the *de novo* DNA methyltransferases, DNMT3A and DNMT3B. Disturbances in expression levels of these miRNAs have been linked to tumorigenesis and tumor aggressiveness; in fact, miR-29 can repress DNA methylation, but is also able to suppress tumorigenesis by preserving the DNA methylation status. In this study, we demonstrate that members of the miR-29 family repress two important components of the DNA demethylation pathway, namely TET1 and TDG. Therefore, miR-29 represses the activities of both DNA methyltransferases and DNA demethylases, which have opposing functions in the control of DNA methylation (Figure 6). In accordance with its proposed competing roles, miR-29 upregulated DNA methylation in PC9 cells and downregulated methylation in A549 cells (Figure 5), suggesting that miR-29 suppresses tumorigenesis by protecting against changes in the existing DNA methylation status and by acting as a stabilizer of DNA methylation. Reduced expression of miR-29 is related to cancer metastasis [15], colon cancer recurrence [16], shorter survival rates of mantle cell lymphoma patients [17] and lung cancer progression [18,19]. Therefore, reduced expression of miR-29 could destabilize the DNA methylation status, leading to aberrant methylation and subsequent tumorigenesis. In fact, A549 had lower miR-29s expression level and lower basal global DNA methylation level (data not shown) compared to PC9, suggesting that a lower miR-29s expression level would destabilize global DNA methylation level.

## 3. Experimental Section

### 3.1. Cell Culture

A549 and PC9 human lung adenocarcinoma-derived cell lines were cultured in MEM medium containing 10% FBS or RPMI1640 medium containing 10% FBS, respectively.

### 3.2. Construction of Luciferase Reporter Plasmids

DNA fragments containing the 3′-UTRs of TET1, TDG, DNMT3A and DNMT3B were amplified by PCR using primers containing XbaI or FseI restriction sites. Amplified fragments were cloned into the corresponding sites in the 3′-UTR of the luciferase reporter vector, pGL3-Control (Promega, Madison, WI, USA). The primer sequences were as follows: TET1 sense, GCT CTA GAG CCC TAT AAC CAT TGG GTC T; TET1 antisense, GGG GCC GGC CTG AAG CAG CTG AAG CAA TAA AC; TDG sense, GCT CTA GAC AGC CCC ATA AGA TTC CAG A; TDG antisense, GGG GCC GGC CTG ATG CAA GGC ACT TCA AA; DNMT3A sense, GCT CTA GAC GAA AAG GGT TGG ACA TCA T; DNMT3A antisense, GGG GCC GGC CGC CGA GGG AGT CTC CTT TTA; DNMT3B sense, GCT CTA GAC TGA CTC TTG CAG GGG TAG C; and DNMT3B antisense, GGG GCC GGC CGT TAC GTC GTG GCT CCA GTT.

### 3.3. Reporter Assay

Luciferase reporter plasmids were transiently transfected into A548 and PC9 cells using Lipofectamine 2000 (Invitrogen, Grand Island, NY, USA), according to the manufacturer’s protocol. Briefly, cells seeded into a 24-well tissue culture dish were exposed to transfection mixtures containing 0.1 μg of luciferase reporter plasmid, 0.05 μg of pRL-TK control vector (Promega) and 10 pmol of miRNA. Cells were harvested 48 h after transfection. Luciferase assays were performed according to the manufacturer’s protocol (Promega). The pRL-TK plasmid was used to normalize firefly luciferase activity to Renilla luciferase activity, to correct for transfection efficiency. Control miRNA and miR-29 were purchased from B-Bridge (Tokyo, Japan).

### 3.4. Transfection of Cells with miRNA

Transient transfections of A548 and PC9 cells with miRNAs were performed using Lipofectamine 2000 (Invitrogen), according to the protocol recommended by the manufacturer. Briefly, cells seeded into a 6-well tissue culture dish were transfected with 100 pmol of miRNA. Cells were harvested 48 h after transfection. Transfected miRNAs are mature miRNA mimic molecules. DNA and RNA species were extracted and were subjected to LUMA and quantitative RT-PCR assays, respectively. The primer sequences used for quantitative RT-PCR were as follows: TET1 sense, CCG AAT CAA GCG GAA GAA TA; TET1 antisense, TAA AAT GGG GTT CGG TTT CA; TDG sense, AGG AGC TTC AGC CAT CAG TT; TDG antisense, GAA TGG AAG CGG AGA ACG; DNMT3A sense, ATA AGC TGG AGC TGC AGG AG; DNMT3A antisense, TGA AGA CAG GAA AAT GCT GGT; DNMT3B sense, ATG AAG GTT GGC GAC AAG AG; and DNMT3B antisense, CCC TGT GAG CAG CAG AAA CT; ACTB sense, GAT GCA GAA GGA GAT CAC TGC; and ACTB antisense, GTA CTT GCG CTC AGG AGG AG.

### 3.5. LUMA Assay

LUMA assays were performed as described previously [21]. Briefly, genomic DNA (300–500 ng) was cleaved with HpaII (45 U) and EcoRI (25 U) or MspI (45 U) and EcoRI (22.5 U) in two separate 15 μL reactions containing 33 mM Tris-acetate (pH 7.9), 10 mM Mg-acetate, 66 mM K-acetate and 0.1 mg/mL BSA. The reactions were incubated at 37 °C for 4 h, and then, 15 μL of annealing buffer (20 mM Tris-acetate (pH 7.6) and 2 mM Mg-acetate) was added. Samples were placed in a PyroMark 24 pyrosequencing system (Qiagen, Valencia, CA, USA), and the instrument was programmed to add dNTPs in four consecutive steps: Step 1, dATP (the derivative dATPαS was used, because it does not react directly with luciferase and, hence, prevents the generation of non-specific signals); Step 2, mixture of dGTP and dCTP; Step 3, dTTP; and Step 4, mixture of dGTP and dCTP. Peak heights were calculated using the PyroMark 24 software. The HpaII/EcoRI and MspI/EcoRI ratios were calculated as (dGTP + dCTP)/dATP. The HpaII/MspI ratio was defined as (HpaII/EcoR1)/(MspI/EcoRI). The global methylation ratio was calculated as one minus the HpaII/MspI ratio.

### 3.6. Quantitative Methylation-Specific PCR

Quantitative PCR was performed using bisulfite-converted genomic DNA. Primers specific to methylated RASSF1 DNA were as follows: RASSF1 sense, TTA GCG TTT AAA GTT AGC GAA GTA C; and RASSF1 antisense, ATA AAC TCA AAC TCC CCC GAC.

## 4. Conclusions

Members of the miR-29 family repress the activities of both DNA methyltransferases and DNA demethylases, which have opposing functions in the control of DNA methylation. The results presented in this study demonstrate that the miR-29 family directly represses DNA methyltransferases, as well as TET1 and TDG, which are two major factors involved in DNA demethylation. These findings suggest that miR-29 suppresses tumorigenesis by protecting against changes in the existing DNA methylation status, rather than by preventing *de novo* methylation.

## Supplementary Information
