# Supplementary material for: miR-29 Represses the Activities of DNA Methyltransferases and DNA Demethylases
Source: Int J Mol Sci. 2013 Jul 12;14(7):14647–58. doi: 10.3390/ijms140714647 (PMC3742264; doi:10.3390/ijms140714647)
Supplement: Supplementary file 1 [file ijms-14-14647-s001.pdf]

Supplementary Information

Figure S1. Members of the miR-29 family impact endogenous TET1 protein. Western blots of TET1 were performed 48 h after transfection of A549 or PC9 cells with miR-29 or control miRNA.

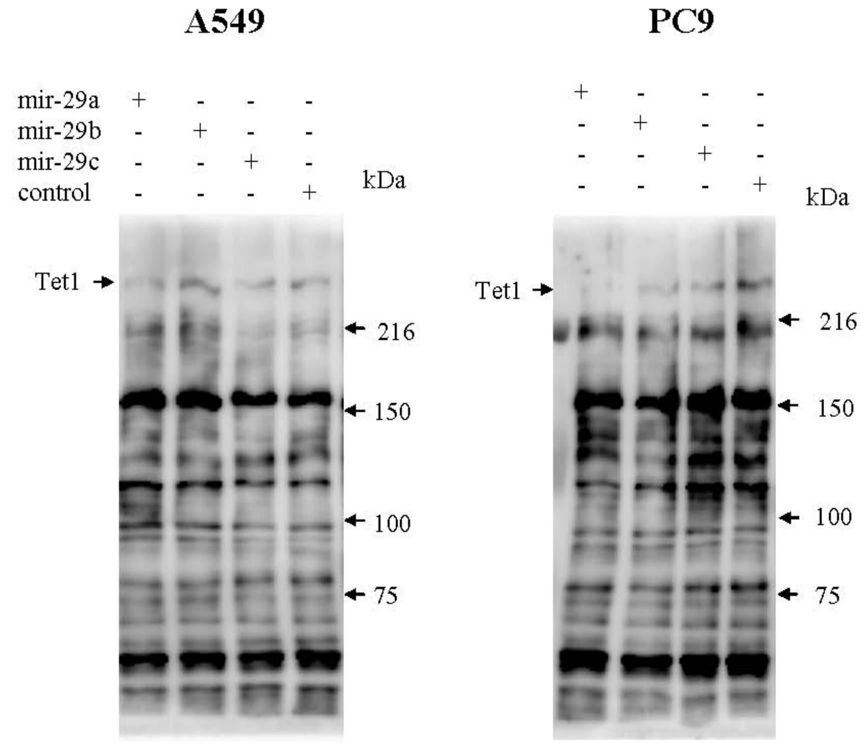

Figure S2. Members of the miR-29 family impact endogenous TDGprotein. Western blots of TDG were performed 48 h after transfection of A549 or PC9 cells with miR-29 or control miRNA.

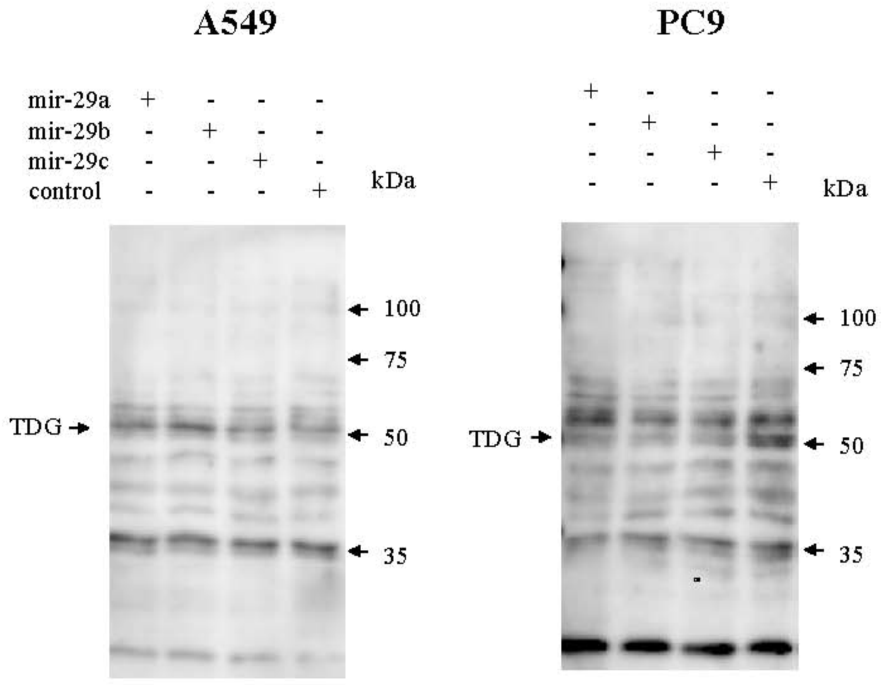

Figure S3. Members of the miR-29 family impact endogenous DNMT3Aprotein. Western blots of DNMT3A were performed 48 h after transfection of A549 or PC9 cells with miR-29 or control miRNA.

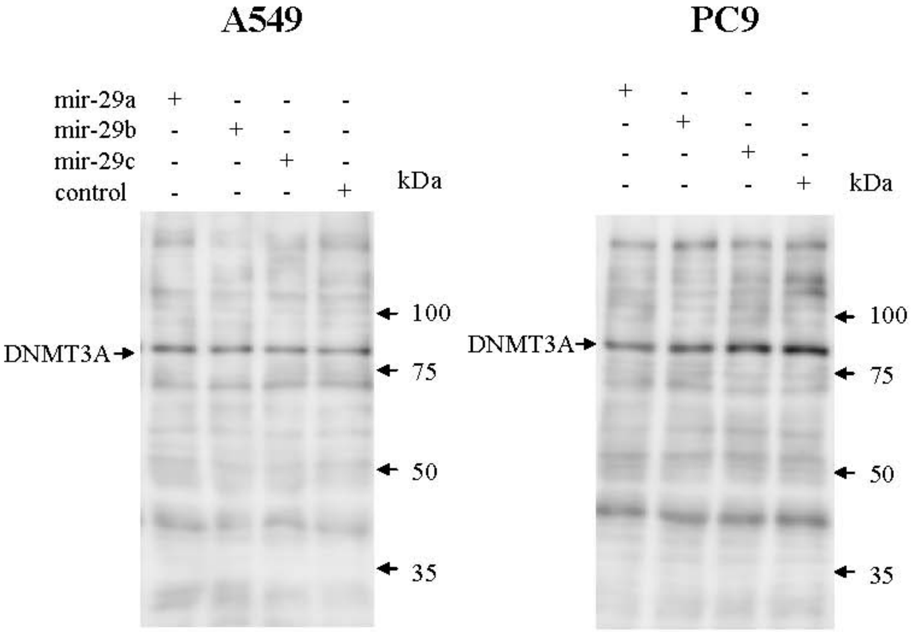

Figure S4. Members of the miR-29 family impact endogenous DNMT3Bprotein. Western blots of DNMT3B were performed 48 h after transfection of A549 or PC9 cells with miR-29 or control miRNA.

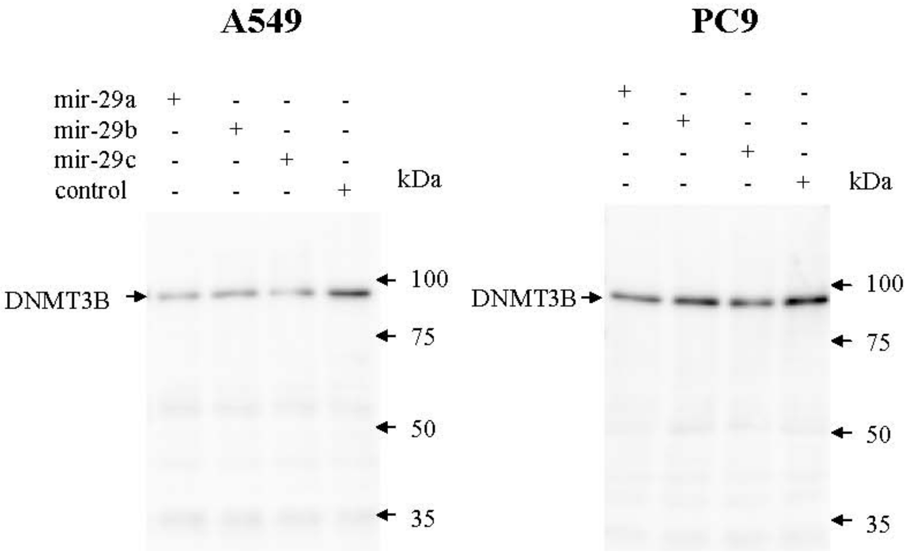

Figure S5. Western blots of control tubulinwere performed 48 h after transfection of A549 or PC9 cells with miR-29 or control miRNA.

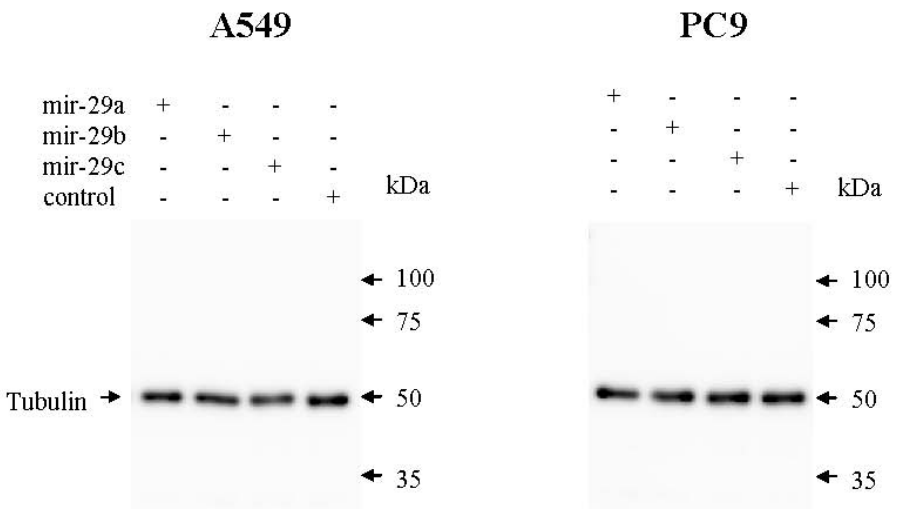

Figure S6. Members of the miR-29 family impact endogenous targets. Band intensity of the Western blot of TET1, TDG, DNMT3A and DNMT3B performed 48 h after transfection of A549 or PC9 cells with miR-29 or control miRNA.

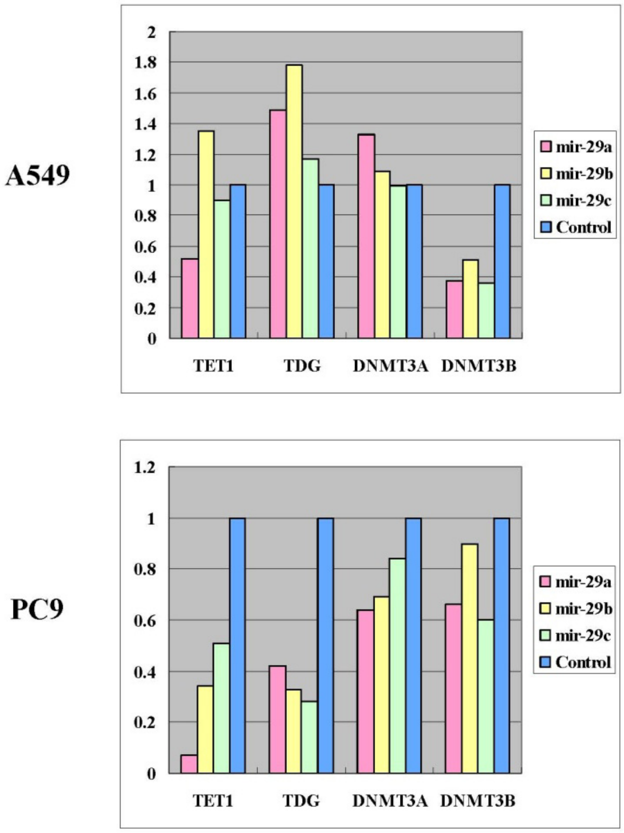

© 2013 by the authors; licensee MDPI, Basel, Switzerland. This article is an open access article distributed under the terms and conditions of the Creative Commons Attribution license (http://creativecommons.org/licenses/by/3.0/).
